# Smoking, longer disease duration and absence of rhinosinusitis are related to fixed airway obstruction in Koreans with severe asthma: findings from the COREA study

**DOI:** 10.1186/1465-9921-12-1

**Published:** 2011-01-03

**Authors:** Taehoon Lee, Yoon Su Lee, Yun-Jeong Bae, Tae-Bum Kim, Seon Ok Kim, Sang-Heon Cho, Hee-Bom Moon, You Sook Cho

**Affiliations:** 1 Department of Allergy and Clinical Immunology, Asan Medical Center, University of Ulsan College of Medicine, Asanbyeongwon-gil 86, Songpa-gu, Seoul, 138-736, Korea; 2 Department of Biostatistics, Asan Medical Center, University of Ulsan College of Medicine, Asanbyeongwon-gil 86, Songpa-gu, Seoul, 138-736, Korea; 3 Department of Asthma, Allergy and Clinical Immunology, Department of Internal Medicine, Seoul National University College of Medicine, Seoul, 110-744, Korea

## Abstract

**Background:**

The clinical manifestations of severe asthma are heterogeneous. Some individuals with severe asthma develop irreversible fixed airway obstruction, which is associated with poor outcomes. We therefore investigated the factors associated with fixed airway obstruction in Korean patients with severe asthma.

**Methods:**

Severe asthma patients from a Korean adult asthma cohort were divided into two groups according to the results of serial pulmonary function tests. One group had fixed airway obstruction (FAO) [forced expiratory volume in 1 second (FEV1)/forced vital capacity (FVC) ratio < 0.7, n = 119] and the other had reversible airway obstruction (RAO) [FEV1/FVC ratio ≥ 0.7, n = 116]. Clinical and demographic parameters were compared between the two groups.

**Results:**

Multivariate analysis showed that longer duration of disease, greater amount of cigarette smoking and absence of rhinosinusitis were significantly related to the development of FAO in severe asthmatics. Other parameters, including atopic status, pattern of airway inflammatory cells in induced sputum, and frequency of asthma exacerbations did not differ between the FAO and RAO groups.

**Conclusion:**

Severe asthma patients with longer disease duration and the absence of rhinosinusitis are more likely to develop FAO. This study also demonstrates the importance of quitting smoking in order to prevent irreversible airway obstruction. Further investigation is required to determine the mechanism by which these factors can modify the disease course in Korean patients with severe asthma.

## Introduction

Asthma, as a chronic airway inflammatory disease, is characterized by recurrent episodes of symptomatic reversible airflow obstruction and airway hyperreactivity to nonspecific stimuli. The early use of inhaled corticosteroids, which reduce airway inflammation and improve pulmonary function, is remarkably effective to control asthma symptoms in these patients[1,2]. However, a subset of asthmatic patients does not respond well to inhaled corticosteroids; most of these patients therefore show uncontrolled asthma symptoms despite treatment with anti-asthmatic medications. Although only 5% of individuals with asthma have severe asthma, they suffer from associated morbidities and show poor quality of life. Furthermore, almost 50% of the healthcare costs for all asthma patients were spent for the treatment of severe asthma [3]. Treatment of severe asthma, therefore, has become a major challenge for physicians.

The effective management of severe asthma requires a thorough understanding of its pathobiology. Clinical manifestations in patients with severe asthma can vary widely [4,5]. Among the clinical characteristics of severe asthma are its progression to irreversible airway obstruction and its poor response to glucocorticoid therapy. Some severe asthma patients show a progressive loss of lung function, which may be related to fixed airway obstruction (FAO). In contrast, other severe asthma patients maintain pulmonary function. FAO has been reported in up to 50% of patients with severe asthma [6]. FAO has also been associated with poor long-term prognosis since patients with this phenotype show a continuous decline in lung function and a high frequency of asthma exacerbations [7].

Modification of factors causing FAO may be critical in preventing morbidities in severe asthmatics with FAO. Among the patient characteristics found to be risk factors for FAO are advanced age, male sex, smoking, longer asthma duration, aspirin sensitivity and sputum eosinophilia [6,8]. Asthma is a multifactorial disorder, with clinical features affected by both genetic and environmental factors. Thus, the risk factors related to FAO in Korean patients with severe asthma may differ from factors associated with FAO in patients from other countries. To date, however, the clinical characteristics of severe asthma patients with FAO in Korea have not been reported. We therefore assessed the clinical features of patients with severe asthma and FAO in an adult asthma cohort recently analyzed in Korea [9]. To determine the clinical factors associated with FAO, we compared the clinical features in severe asthma patients with FAO with those in severe asthma patients with reversible airway obstruction (RAO).

## Materials and methods

### Study subjects

The COREA (*CO*hort for *R*eality and *E*volution of adult *A*sthma in Korea) is a prospective, observational, multicenter cohort of Korean adults with asthma designed to investigate the characteristics of Korean asthma patients [9]. The study subjects are 14 years or older and have been suffering from at least one of the chronic, persistent, respiratory symptoms of dyspnea, cough, sputum production or wheezing for more than three months. Asthma was then diagnosed by demonstration of airway reversibility or bronchial hyperresponsiveness (BHR). Airway reversibility consisted of an improvement in FEV_1 _of at least 12% post-bronchodilator (200 mcg of albuterol by means of a metered-dose inhaler), or 20% or more over time or after corticosteroid treatment. BHR was defined as PC20 (provocation concentration that caused a decrease in FEV1 of 20%) of methacholine ≤ 16 mg/mL before asthma treatment [10]. In order to exclude smoking-related COPD, patients with apparent emphysema based on simple chest radiography were excluded and every asthma subject was diagnosed by experienced clinicians in the field of allergy or pulmonology at each institution.

### Inclusion criteria of this study

A total of 1,520 asthma patients were registered by the 11 centers; of these, 352 patients were classified as severe asthma by the clinicians and finally 235 patients were selected for the study. Detailed criteria for the enrollment were as follows: 1) all patients should have one of the following clinical components including an FEV1 less than 60% of the predicted value, daily symptoms, frequent exacerbations, frequent nocturnal symptoms, and limited physical activity (based primarily on Global Initiative for Asthma (GINA) 2002 guidelines [2]); 2) requirement for treatment should be moderate- to high-dose ICS and an LABA combination inhaler with or without sustained-release theophylline, leukotriene modifier, oral ß2 agonist or oral glucocorticoids; 3) the patients should undergo all 3 pulmonary function tests under stable conditions without asthma exacerbation at the time of enrollment (baseline) and at 3 and 12 months after enrollment. Among those 3 pulmonary function tests, at least one test was performed both before and after inhalation of 200 microgram of albuterol on top of ICS and LABA; 4) all the patients were believed to be adequately treated and adherent to prescribed medications.

### Exclusion criteria of the study

**Figure 1:**
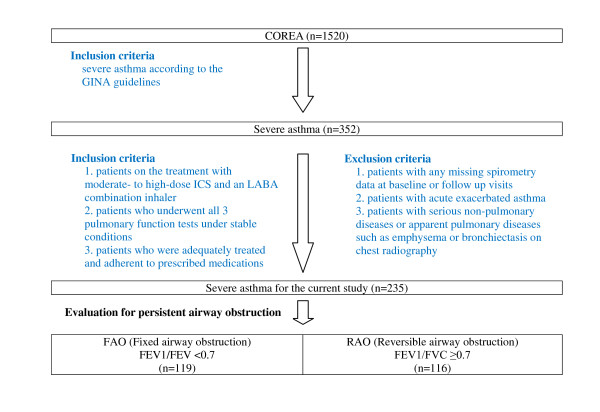
**Flowchart showing selection of patients with severe asthma**.

Patients with acute exacerbated asthma or any missing spirometry data among the 3 visits (baseline and 2 follow-up visits) were excluded. Patients with any of the following conditions were also excluded; 1) serious non-pulmonary diseases such as heart failure, cancers and severe psychiatric disorders; 2) other pulmonary diseases such as apparent emphysema, bronchiectasis, or destroyed lung caused by previous medical conditions like pulmonary tuberculosis on chest radiography. Figure 1 summarizes these inclusion/exclusion criteria.

### Definition of fixed airway obstruction and reversible airway obstruction

Patients with severe asthma were classified into two groups, those with FAO and those with RAO, based on the results of their pulmonary function tests. FAO was defined as an FEV1/FVC ratio < 0.7 on all of three pulmonary function tests despite use of high-dose inhaled corticosteroids (ICS) and long-acting β2-agonists (LABA)[8,11]. RAO was defined when an FEV1/FVC ratio > 0.7 was proved on at least one pulmonary function test.

### Study design

Demographic and clinical data recorded at the time of enrollment included asthma duration, age at asthma onset, the number of episodes of asthma exacerbation, presence of rhinosinusitis, atopic status and results of laboratory tests. Pulmonary function tests were performed at the time of enrollment and 3 and 12 months later. Baseline methacholine bronchial provocation tests were performed in some patients (33 in FAO and 32 in RAO, respectively) with PC20, being an indicator of bronchial hyperresponsiveness (BHR).

The episodes of exacerbation, emergency department visits, and hospitalizations were determined based on the patients' responses to questions such as, 'Have you ever visited any hospital or emergency room or ever been hospitalized because of suddenly worsening asthmatic symptoms, such as dyspnea and wheezing?'. In addition, the presence of rhinosinusitis was defined according to the patients' medical history in a simple questionnaire which included the questions: 'Have you ever been diagnosed with chronic allergic rhinosinusitis?' and 'Have you recently had one or more symptoms of nasal congestion, including runny nose, sneezing, nasal itching, and post-nasal drip?'. Rhinoscopy or radiologic studies were not routinely performed.

A skin prick test was conducted using the common inhalant allergens in Korea, including *Dermatophagoides pteronyssinus*, *Dermatophagoides farinae*, tree pollen mixtures, grass pollen mixtures, ragweed, mugwort, cat fur, dog fur, cockroach, aspergillus, alternaris, and other fungus mixtures (Allergopharma, Darmstadt, Germany), and if any positive response was found in the skin test, we defined the subject as having atopy.

In our study, the induced sputum analysis was conducted at enrollment according to the method previously described [12]. In brief, sputum induction was performed by inhalation of hypertonic saline (NaCl 4.5%) for 15 min after premedication with inhaled albuterol. The sputum was treated with dithiothreitol and phosphate buffered saline and was then centrifuged at 1,000 rpm for 10 min. Cells were stained with Diff-Quik (American Scientific Products, McGaw, IL, U.S.A.).

Methacholine bronchial provocation test was performed using a dosimeter-controlled jet nebulizer with increasing doses of methacholine from 0.625 to 25 mg/mL. Each dose of methacholine was inhaled during five, slow and vital-capacity breaths. At one and three minutes after each set of inhalations, spirometry was recorded. The interval between one dose and the next was five minutes.

To assess the factors linked to the development of FAO, all parameters were compared between the FAO and RAO groups. To determine whether the 235 patients analyzed were representative of the entire cohort of 352 subjects with severe asthma, the clinical characteristics of the 117 unselected and 235 selected patients were compared. The study protocol was approved by the institutional review boards of all participating hospitals. (Asan Medical Center, University of Ulsan; Seoul National University Hospital; Bundang Seoul National University Hospital; Yonsei University Hospital; Chungang University Hospital; Ewha Womans University Hospital; Hanyang University Hospital; Soonchunhyang University Hospital; Ajou University Hospital; ChonBuk National University Hospital).

### Statistical methods

Fisher's exact tests and χ^2 ^tests were used to compare categorical variables and independent Student's *t-*tests were used to compare continuous variables. The Mann-Whitney *U*-test was also used to compare continuous variables that did not follow a normal distribution. Each variable was assessed in the univariate analyses. Variables showing a significant correlation (p ≤ 0.1) were included in the multivariate logistic regression analysis. Smoking (≥ 5 pack-years vs < 5 pack-years)[13,14], asthma duration (≥ 15 years vs < 15 years)[15], rhinitis, patient age (≥ 50 years old vs < 50 years old) [16,17], age at onset (≥ 40 years old vs < 40 years old)[18], and gender and atopic status which were selected for identification of the independent factors associated with FAO. Statistical analyses were performed using the SPSS statistical software package (version 18). *P *< 0.05 was considered statistically significant.

## Results

Of 235 patients with severe asthma, 119 (51%) had FAO and 116 had RAO (Figure 1). The baseline characteristics of the two groups are shown in Tables 1, 2 and 3.

**Table 1:** Demographic data of patients with severe asthma

Characteristics	FAO (n = 119)	RAO (n = 116)	P-value
Age, yr	58.3 ± 12.8	47.1 ± 14.3	<0.001
Male, %	64	40	<0.001
BMI, kg/m^2^	23.4 ± 2.9	23.9 ± 3.5	0.239
Duration of asthma, yr	10 [4 - 18]	6 [3 - 12]	0.083
Onset age, yr	47.5 ± 15.4	39.7 ± 15.6	<0.001
Treatment duration, yr	5 [2 - 10]	5 [3 - 10]	0.851
Numbers of ER visit due to asthma	2 [1 - 5]	2 [1 - 5]	0.751
Numbers of admission due to asthma	2 [1 - 5]	1 [1 - 4]	0.070
Presence of exacerbation, %	51	58	0.339
Presence of atopy, %	34	49	0.068
Presence of rhinosinusitis, %	50	66	0.014
Duration of smoking, pack-years	30 [18.7 - 40]	14 [4.5 - 25]	<0.001
Smoking, current/ex/non, n	15/61/24	14/42/44	0.003

Abbreviations:

FAO, fixed airway obstruction; RAO, reversible airway obstruction; ER, emergency room

Data are presented as mean ± SD, % or median [interquartile range].

*P*-values were determined using the Mann-Whitney *U*-test, chi-square or Fisher's test.

**Table 2:** Pulmonary function tests of patients with severe asthma

Characteristics	FAO (n = 119)	RAO (n = 116)	P-value
FEV1, initial, predicted %	54.8 ± 19.9	74.3 ± 22.5	<0.001
FEV1/FVC, initial, predicted %	56.3 ± 11.1	77.6 ± 10.6	<0.001
FEV1/FVC, 3 months after enrollment, predicted %	57.2 ± 10.6	80.0 ± 6.7	<0.001
FEV1/FVC, 12 months after enrollment, predicted %	58.9 ± 9.6	80.9 ± 7.1	<0.001
Baseline PC20, mg/dL*	2.7 ± 4.1	5.6 ± 6.4	0.036

*Baseline methacholine bronchial provocation tests were done in 65 subjects:

33 patients with FAO (n = 119), 32 patients with RAO (n = 116)

Abbreviations:

FAO, fixed airway obstruction; RAO, reversible airway obstruction; FEV1, forced expiratory volume in 1 second; FVC, forced vital capacity; PC20, provocation concentration that caused a 20% decrease in FEV1

Data are presented as mean ± SD.

**Table 3:** Laboratory data in patients with severe asthma

Characteristics	FAO (n = 119)	RAO (n = 116)	P-value
Blood neutrophils,/uL	5161.3 ± 2807.0	4833.4 ± 2493.4	0.514
Blood eosinophils,/uL	320.7 ± 345.8	378.6 ± 373.9	0.468
Blood neutrophils, %	58.8 ± 14.4	57.0 ± 12.4	0.327
Blood eosinophils, %	4.1 ± 4.3	4.9 ± 5.2	0.308
Sputum neutrophils, %*	29.1 ± 24.5	29.2 ± 25.6	0.780
Sputum eosinophils, %*	16.4 ± 21.2	8.8 ± 14.3	0.168
Uric acid, mg/dL	5.4 ± 1.5	5.1 ± 1.6	0.500
CRP, mg/dL	0.8 ± 2.5	0.3 ± 0.7	0.170
Log IgE	2.1 ± 0.6	2.2 ± 0.6	0.475

*Successful sputum counts were done in 64 subjects:

37 patients with FAO (n = 119), 27 patients with RAO (n = 116)

Abbreviations:

FAO, fixed airway obstruction; RAO, reversible airway obstruction; CRP, C-reactive protein

Data are presented as mean ± SD.

Patients with FAO were significantly older than those with RAO (58.3 ± 12.8 years vs 47.1 ± 14.3 years, p < 0.001). In addition, patients with FAO were more likely to be male (64% vs 40%, p < 0.001), have a later age of asthma onset (47.5 ± 15.4 years vs 39.7 ± 15.6 years, p < 0.001) and have a longer smoking history (30 [18.7 - 40] pack-years vs 14 [4.5 - 25] pack-years, p < 0.001). Symptoms of rhinosinusitis were significantly less common in patients with FAO than in patients with RAO (50% vs 66%, p = 0.014). There were no significant between-group differences in treatment duration, numbers of emergency department visits and admissions due to acute asthma exacerbation, BMI (kg/m^2^), atopic status and history of asthma exacerbation (Table 1).

The initial percent predicted FEV1 was significantly lower in the FAO than in the RAO group (54.8 ± 19.9% vs 74.3 ± 22.5%, p < 0.001), as were initial (56.3 ± 11.1% vs 77.6 ± 10.6%, p < 0.001) and best (58.9 ± 9.6% vs 80.9 ± 7.1%, p < 0.001) FEV1/FVC ratios after treatment (3 or 12 months after enrollment) and PC20 values (2.7 ± 4.1 mg/dL vs 5.6 ± 6.4 mg/dL, p = 0.036) (Table 2).

We found that there were no between-group differences in serum concentrations of total IgE, CRP and uric acid. Complete blood cell analysis showed no significant between group differences in neutrophil and eosinophil counts, and there were no differences in induced sputum analysis (Table 3).

Multivariate analysis identified several independent factors associated with FAO (Table 2). Smoking was the most potent independent factor associated with FAO, with an adjusted odds ratio (OR) for smoking more than 5 pack-years of cigarettes of 6.6 [95% confidence interval (CI), 1.4 to 30.1]. In addition, FAO was associated with long standing (> 15 years) asthma (adjusted OR, 5.3; 95% CI, 1.2 to 23.8) and an absence of rhinosinusitis (adjusted OR, 3.8; 95% CI, 1.2 to 12.1). Advanced age, late-onset asthma, presence of asthma exacerbation history, male sex and presence of atopy were not independently associated with the development of FAO in multivariate analysis (Table 4).

**Table 4:** Factors associated with fixed airway obstruction in patients with severe asthma

	Univariate analysis		Multivariate analysis	
		
	p-value	Crude OR (95% CI)	p-value	Adjusted OR (95% CI)
Smoking more than 5 pack-years	< 0.001	3.8 (2.1-7.0)	0.015	6.6 (1.4-30.1)
Asthma more than 15 years	0.064	1.8 (1.0-3.4)	0.028	5.4 (1.2-24.4)
Absence of rhinosinusitis	0.014	2.0 (1.2-3.4)	0.023	3.8 (1.2-12.3)
Age ≥50 years	< 0.001	3.2 (1.9-5.6)	0.632	1.6 (0.2-10.4)
Onset age < 40 years	0.018	0.5 (0.3-0.9)	0.893	1.1 (0.2-8.2)
Male sex	< 0.001	2.7 (1.6-4.7)	0.907	1.1 (0.3-4.9)
Presence of atopy	0.069	0.5 (0.2-1.1)	0.097	0.4 (0.1-1.2)

Abbreviations: OR, odds ratio; CI, confidence interval

When we compared the 235 study subjects and the 117 patients with severe asthma who were not enrolled in this study, we found that the proportion of subjects with rhinosinusitis was lower in selected than in non-selected patients (58% vs 72%, p = 0.021) and that age at asthma onset was higher in selected than in non-selected patients (43.7 ± 16.0 years vs 40.1 ± 16.3 years, p = 0.037). Other clinical and demographic characteristics, however, did not differ significantly between the two groups (data not shown).

## Discussion

We have shown here that longer asthma duration and the absence of rhinosinusitis, in addition to cigarette smoking, are independent factors associated with the development of irreversible FAO in Korean patients with severe asthma. Although effective methods to improve the management of patients with severe asthma remain a challenge for physicians, the results presented here provide further evidence of the importance of quitting smoking in preventing irreversible airway obstruction, especially in patients with severe asthma [19]. These results also indicate that patients with a certain subtype of severe asthma, without upper airway pathology such as rhinosinusitis, are more likely to develop irreversible airway obstruction. Moreover, our findings highlight the significance of understanding the heterogeneity of severe asthma and suggest that the pathogenesis of severe asthma without rhinosinusitis might differ from that of severe asthma with rhinosinusitis. Clarification of the mechanisms underlying certain subtypes of severe asthma may provide clues to therapeutic strategies for overcoming this condition.

Although only a small proportion of patients with asthma have severe asthma, this subtype of asthma is associated with most asthma-related morbidities and asthma-associated costs [3]. Severe asthma is characterized by frequent asthma attacks, resistance to high-dose ICS treatment and fixed irreversible airway obstruction [20]. As many studies have suggested that the clinical manifestations of severe asthma are heterogeneous [4,5], progressive decline in lung function leading to FAO has been reported in about 50% of patients with severe asthma, which is in agreement with our and other results [6]. FAO is thought to be closely related to the development of airway remodeling, as well as with poorer prognosis [21,22]. Identification of risk factors related to the occurrence of FAO in severe asthma may therefore be critical because it may provide a foundation for clarifying the pathogenesis of the disease and may ultimately lead to effective therapeutic approaches. Although several studies had assessed the factors causing irreversible airway obstruction in asthmatic patients of other ethnic groups, the characteristic features of severe asthma with FAO and the factors associated with its pathogenesis had not previously been clarified in Korean patients with severe asthma.

Various clinical characteristics have been proposed as risk factors for the development of FAO in patients with severe asthma [19]. Several studies have found that smoking is one of the most important risk factors for FAO [15,23]. Other suggested risk factors for FAO include adult-onset asthma, airway hyperresponsiveness and, most importantly, sputum eosinophilia [6]. Low lung function at a young age has been reported to be a risk factor for FAO, suggesting that structural changes already occur during the early course of the disease [24]. Other possible risk factors for FAO include recurrent severe asthma exacerbation, higher degree of asthma severity, male sex [8,25-27], longer disease duration [28], advanced age and aspirin sensitivity [8]. In agreement with these findings, we found that longer disease duration and smoking history were closely associated with FAO in Koreans with severe asthma.

In the current study, we also found that an absence of rhinosinusitis was closely associated with FAO in our patient population. Interestingly, the factor of absence of rhinosinusitis had not been previously described as a risk factor for FAO. Rather, patients with severe asthma were reported to have more severe rhinosinusitis than were those with mild asthma [1]. Furthermore, uncontrolled rhinosinusitis is a well known compounding factor which is involved with poorly controlled asthma [29]. Meanwhile, there is a report that the prevalence of rhinosinusitis has been found to decrease, and asthma severity to increase, with age [30]. Our finding, of a reduced prevalence of rhinosinusitis in patients with more severe asthma, may be due to the spontaneous regression of rhinosinusitis during the course of asthma. The relationship between FAO and the absence of rhinosinusitis in patients with severe asthma, however, is not yet clear, although it may represent a unique feature of Korean patients with severe asthma.

In the present study, asthmatic patients with FAO tended to be more non-atopic, although the difference was not statistically significant. Non-atopic asthma without rhinosinusitis may be a pre-requisite factor for the development of FAO in our patient cohort, because atopic asthma is usually milder, has an earlier onset and is more commonly accompanied by other allergic disease such as allergic rhinosinusitis [1,31,32]. Classification of severe asthma into several groups through cluster analysis also found that one cluster was characterized by childhood onset, more atopic and presence of RAO, while the other cluster consisted of patients with later-onset disease, less atopic and FAO[4]. Thus, FAO in severe asthma may be related to the absence of rhinosinusitis due to the non-atopic conditions of the subjects.

Many studies have reported that longer duration of asthma may lead to irreversible airway obstruction [28,33,34]. Prolonged airway inflammation may be related to increased remodeling of the airway walls and a greater reduction in the airway lumen over time. We also observed significant correlations between asthma duration and FAO in multivariate analysis.

In previous reports, advanced age was associated with the development of FAO [33,35]. Elderly asthma patients tend to show reduced immediate bronchodilator responses and decreased reversibility of airway obstruction after treatment. These results may be caused by the occurrence of irreversible airway remodeling in elderly asthma patients, who presumably have longer disease duration. However, in our study, older age was not an independent risk factor for FAO.

Sputum eosinophilia is thought to be an important marker of FAO, suggesting that eosinophilic airway inflammation may contribute to persistent airflow limitation in patients with severe asthma[36,37]. Eosinophils can secrete a variety of chemical mediators which may induce airway remodeling [36,37]. We found, however, no significant differences between the FAO and RAO groups in numbers of sputum or blood eosinophils, although the numbers tended to be higher in the patients with FAO.

This study had several limitations. First, as it was observational study in design, the clinical situations of the patients may have been diverse regarding types of medications, adherence to medications, familiarity with a certain inhaler device, and environmental control of workplaces or home. These might have affected the clinical course and the proportion of FAO, although all enrolled patients were optimally treated by experts and with correct medications and were also believed to be compliant with their prescribed medications. In addition, FAO was defined one year after enrollment, a relatively short follow-up period. FAO may have been a consequence of long lasting inflammatory processes in the asthmatic airways. Thus, a longer-term follow-up study is required to determine the risk factors for FAO in patients with severe asthma.

Another limitation of our study is that the presence or absence of rhinosinusitis was basically identified by history-taking and according to patients' symptoms. As not all study patients underwent objective measures to confirm their diagnosis, such as rhinoscopy, paranasal sinus radiography or ostiomeatal unit computed tomography, patients with asymptomatic rhinosinusitis may have been misclassified in this study. Future studies should include objective tests to exclude patients with asymptomatic rhinosinusitis.

As it is not always possible to completely exclude smoking-related COPD, this may be another important limitation of this study. Although patients whose major diagnosis could be COPD were not included in this study, some of the participants may have belonged to the asthma and COPD overlap syndrome population [38]. Moreover, not only the patients with RAO but also those with FAO may be heterogeneous in terms of the reversibility according to the level of airway obstruction. Although in the current study FAO was defined as existing only when all three pulmonary function tests, when performed under stable conditions, showed an FEV1/FVC ratio < 0.7, considerable heterogeneity in the patients clinical features is still present even in severe asthma. Thus, there is an urgent need to clarify the heterogeneity of asthma, and further studies should be conducted.

Finally, our population of patients with severe asthma might not be representative of all Korean patients with severe asthma enrolled in the COREA since some clinical features differed between the groups of included and non-included patients. Hence, further studies with larger study populations are also needed to confirm our findings.

In conclusion, this study is the first to report factors that may be related to persistent airflow limitation in Koreans with severe asthma. We found that long-standing asthma and the absence of rhinosinusitis were more likely to lead to persistent irreversible airway obstruction. In addition, our findings further demonstrated that smoking is an important cause of irreversible airway damages. Further studies are required to clarify the mechanisms underlying the development of FAO in Korean subjects with severe asthma.

## Abbreviations

BHR: bronchial hyperresponsiveness; COREA: Cohort for Reality and Evolution of Adult Asthma Korea; CRP: C-reactive protein; FAO: fixed airway obstruction; FEV1: forced expiratory volume in 1 second; FVC: forced vital capacity; ICS: inhaled corticosteroids; LABA: long acting β2-agonists; PC20: provocation concentration that caused a decrease in FEV1 of 20%; RAO: reversible airway obstruction

## Competing interests

The authors declare that they have no competing interests.

## Authors' contributions

TL conducted the clinical assessments, analysed and interpreted data and wrote the first draft; YSL and YJB contributed to data collection and interpretation; TBK advised on and helped with the study design, contributed to data analysis and interpretation; SOK helped with the statistical analysis; HBM contributed to the study methods, advised on research measures, discussed core ideas, commented on data analysis, and helped with data interpretation: SHC contributed to data collection and interpretation; The COREA Study Group contributed to data collection and interpretation; YSC conceived the study and design, advised on and helped with study design, discussed core ideas, designed data collection protocols, and helped with data interpretation and writing. All authors read and approved the final manuscript.
